# Draft genome sequence of *Flavobacterium* sp. ARAG 55.4, an agarolytic bacterium isolated from red algae *Gracilaria tenuistipitata*

**DOI:** 10.1128/mra.00924-25

**Published:** 2025-11-05

**Authors:** Trinh Thi Van Anh, Nguyen Bao Chau, Vu Nguyen Thanh, Nguyen Thanh Thuy

**Affiliations:** 1 Food Industries Research Institute, Hanoi, Vietnam; University of Strathclyde, Glasgow, United Kingdom

**Keywords:** *Flavobacterium*, genome, agar, agarase, sulfatase

## Abstract

The draft genome sequence of *Flavobacterium* sp. ARAG 55.4, an agarolytic bacterium isolated from red algae in Hai Phong, Vietnam, comprises 4,176,422 bp with a G + C content of 34.25%. Genomic annotation revealed 3,559 protein-coding sequences, including 20 putative agarase genes and 30 putative sulfatase genes.

## ANNOUNCEMENT

Agar, the main polysaccharide of red algae, is an important gelling agent in food products. Conventional agar extraction and processing are chemically intensive, whereas agarolytic enzymes from marine bacteria, such as sulfatases and agarases, offer sustainable alternatives for sulfate removal and agarooligosaccharide production (1). We present the genome of *Flavobacterium* sp. ARAG 55.4, which carries multiple predicted sulfatase- and agarase-related genes.

Strain ARAG 55.4 was isolated from red algae collected at a seaweed pond in Hai Phong, Vietnam (20°44.553′N, 106°45.953′E). About 1 g of red algae was transferred into 50 mL M9 containing 0.1% (w/v) agar and incubated at 30°C for 24 h without agitation. The enriched culture was streaked on M9 agar and incubated at 30°C for 48 h. Colonies forming agarolytic zones, indicating agarase activity, were purified by restreaking on M9 agar (2). Sulfatase activity was tested by incubating washed cell suspensions (OD₆₀₀ ≈ 1.0) with 10 mM para-nitrophenol sulfate at 30°C for 24 h; yellow color formation indicated activity (3). Strain ARAG 55.4, positive for both activities, was selected for genome analysis.

*Flavobacterium* sp. ARAG 55.4 was grown aerobically on M9 agar at 30°C for 48 h. Genomic DNA was extracted using the GeneJET Genomic DNA Purification Kit (Thermo Scientific). The 16S rRNA gene was amplified with primers 8F and 1492R (4) and sequenced on ABI 3130xl. Sequence analysis via EzBioCloud (https://www.ezbiocloud.net, update 2025.04.21) showed 98.1% identity to *Flavobacterium flevense* DSM 1076^T^ (jgi.1107673). Genomic DNA was prepared using the NEBNext Ultra DNA Library Prep Kit (NEB) and sequenced on Illumina NovaSeq (2 × 150 bp), yielding 7,366,706 reads. Reads were trimmed with Trimmomatic v0.36 (Q ≥ 30) and quality-checked by FastQC v0.12.1 (5, 6). *De novo* assembly with SPAdes v4.1.0 (7, 8). Genome completeness (99.29%) and contamination (0.08%) were estimated using CheckM v1.2.4 (9) with the *Flavobacteriaceae*-specific marker set (UID2817). Genome annotation used the NCBI Prokaryotic Genome Annotation Pipeline v6.10 (10). Carbohydrate-active enzymes were identified through dbCAN3 (11), and predicted sulfatases were annotated using the SulfAtlas database v2.3.1 (12). Average nucleotide identity (ANI) between strain ARAG 55.4 and *F. flevense* DSM 1076^T^ (GCA_900142775.1), the closest type strain, was computed using FastANI v1.33 (13). Unless otherwise noted, default parameters were used for all software.

The draft genome of strain ARAG 55.4 was assembled into 43 contigs (≥500 bp), with an N50 of 224,363 bp and a maximum contig length of 503,762 bp. The assembly comprises 4,176,422 bp, has a G + C content of 34.25%, and was sequenced to an average depth of 264×. Annotation revealed 3,559 predicted protein-coding sequences, along with 3 rRNA genes and 45 tRNA genes. Among these, twenty putative agarolytic genes were detected, comprising 12 GH16, 1 GH39, 1 GH42, 2 GH86, and 4 GH117 (https://zenodo.org/records/16889182). BLAST analysis against the SulfAtlas database assigned 30 putative sulfatases to the S1 family (formylglycine-dependent sulfatases) (12), distributed across 14 distinct subfamilies (https://zenodo.org/records/16886455). ANI comparison with *F. flevense* DSM 1076ᵀ (GCA_900142775.1) yielded 87.6%, below the recognized species delineation threshold (14), suggesting ARAG 55.4 may represent a novel species within the genus *Flavobacterium*.

## Data Availability

The 16S rRNA gene sequence was deposited in the GenBank under the accession number PV203203. The raw reads and genome assembly of *Flavobacterium* sp. strain ARAG 55.4 are available in GenBank under accession number SRR34900922 and JBPYCT000000000.1, with associated BioSample SAMN50097345 and BioProject PRJNA1294494.
